# A Polyphasic Approach Aids Early Detection of Potentially Toxigenic Aspergilli in Soil

**DOI:** 10.3390/microorganisms7090300

**Published:** 2019-08-29

**Authors:** Giovanni Cafà, Benedetta Caggiano, Michael A. Reeve, Hamzah Bhatti, Sabyan F. Honey, Babar Bajwa, Alan G. Buddie

**Affiliations:** 1 CABI Europe-UK, Bakeham Lane, Egham, Surrey TW20 9TY, UK; 2 CABI CWA, Opposite 1-A, Data Gunj Baksh Road, Satellite Town, Rawalpindi 46300, Pakistan

**Keywords:** *Aspergillus flavus*, aflatoxins, metabarcoding, whole genome sequencing

## Abstract

Key chili and maize growing areas of Pakistan were selected for a focused baseline study of the levels of *Aspergillus* spp. Investigations were undertaken using a combination of molecular and culture-based techniques. Samples investigated included soil samples, one-year-old corn cobs, and fresh chili from selected locations. *Aspergillus* strains obtained from corn cobs were screened using coconut milk agar, resulting in one strain that was positive for aflatoxin production. Whole genome sequencing (WGS) with low coverage techniques were employed to screen the isolates for differences in the ribosomal RNA gene cluster and mitochondrial genome, with the aflatoxigenic strain proving to have a distinctive profile. Finally, strains were subjected to matrix-assisted laser-desorption and ionization time-of-flight mass spectrometry (MALDI-ToF-MS) in order to obtain a proteomic ‘fingerprint’ which was used to distinguish the aflatoxigenic strain from the other isolates. The next generation sequencing (NGS) study was broadened to incorporate metabarcoding with ITS rRNA for determining the microbial biodiversity of the soil samples and presumptive screening for the presence of aflatoxigenic strains. Using information gleaned from the WGS results, a putative aflatoxigenic operational taxonomic unit (OTU) was observed in four of the 15 soil samples screened by metabarcoding. This method may have beneficial applications in early detection and surveillance programs in agricultural soils and commodities.

## 1. Introduction

*Aspergillus flavus* causes a broad spectrum of disease in humans, ranging from hypersensitivity reactions to invasive infections associated with angioinvasion [1]. Together with *A. fumigatus* and *A. terreus*, *A. flavus* is recognized as amongst the most common causes of aspergillosis [2,3,4,5]. In a global context, aflatoxin contamination is a constant concern between the 35N and 35S latitudes, where developing countries are mainly situated. With expanding international trade opportunities, aflatoxin contamination has become a persistent problem for developing countries. The continuing threat from aflatoxin contamination of food, feed, and agricultural commodities to the world population has made aflatoxin research one of the most rapidly developing study areas of food security and public health [6,7,8].

Aflatoxin contamination of agricultural commodities, such as maize, peanuts, almonds, and cottonseed, is a serious risk to human and animal health [9,10,11,12,13]. Aflatoxins are potent carcinogenic and mutagenic compounds produced as secondary metabolites by several *Aspergillus* species, of which *A. flavus* is the most notorious. *A. flavus* belongs to *Aspergillus* section *Flavi* [8] and it is a ubiquitous common soil inhabitant, and is also found in crops and foods at both pre- and post-harvest stages. The fungus overwinters either as mycelium or as resistant structures known as sclerotia. The sclerotia may germinate to produce either hyphae or conidia, which can be further dispersed in the soil and air [1]. Ideal conditions for aflatoxin production include humid and warm weather in combination with injuries on the plant structure (often caused by insect damage [14]). As such, aflatoxins are a major issue in developing countries in Asia and Africa for any temperate to tropical agricultural commodity producer, with significant problems recorded in Kenya [15], Senegal [16], India [17], and Pakistan [18]. Elevated levels of aflatoxins have also been reported from milk in European countries such as Serbia, Croatia, and Romania [19].

Pakistan is an agrarian society where agriculture alone contributes 23% to the total gross domestic product (GDP). Maize is one of the most widely grown cereal crops in the world and has a considerable significance for Pakistan [20]. Pakistan has been reported as the sixth largest exporter of chilies in the world [18], contributing 1.5% to the GDP of Pakistan [21]. Pakistan experiences long, hot, and humid months from March until September, which makes agricultural production highly susceptible to fungal growth. In order to assess the potential of soil to act as a reservoir of infection, a decision was taken to sample in the post-harvest period of the crop calendar, as improper harvesting and drying strategies produce favorable environments for toxin production in chilies.

*A. flavus* is the most prominent cause of mycotoxin-related crop/commodity contamination [22] and therefore warrants particular attention. Strains of *A. flavus* may be split into two main morphotypes that loosely link to their aflatoxin production, as follows: less aflatoxigenic L strains, which produce copious conidia and few large sclerotia, and more aflatoxigenic S strains, which produce relatively few conidia and copious small sclerotia [22,23]. Interestingly, it has been noted that even the non-aflatoxigenic strains have the potential to produce other significant mycotoxins [23]. Amongst the different aflatoxins, the naturally occurring and best-known classes are aflatoxins B1 (AFB1), B2 (AFB2), G1 (AFG1), and G2 (AFG2). Production of these toxin classes differs according to particular species, as follows: *A. flavus* can produce AFB1 and AFB2, whilst *A. parasiticus* may produce both ‘B’ and both ‘G’ toxins [24].

Several molecular techniques and classical morphometric methods have been employed to identify and detect strains belonging to the *Aspergillus* section *Flavi* [25]. The observations of fungal cultures grown on different media are time-consuming and unreliable due to intra- and inter-specific morphological differences. Protein based spectral profiling has been used to discriminate among strains of aflatoxin producers across several species of *Aspergillus* [5,26]. Additionally, chromatographic methods have been employed to discriminate toxin production in strains [10,27]. Several nucleic acid based methods have been employed to classify and detect *Aspergillus* section *Flavi* members, as follows: random amplification of polymorphic DNA (RAPD) [28], amplified fragment length polymorphism (AFLP) [29], microsatellite markers [30] and sequence analyses of the cytochrome b gene, the internal transcribed spacer (ITS) region [31], and the aflatoxin gene cluster [32]. These methods are able to provide important information about the phylogenetic relationships between species but they do not represent a diagnostic method for *A. flavus* [33]. Additionally, a pyrosequencing approach has been employed to enable culture-free quantification of *A. flavus* in cotton crops [34], and quantitative real time PCR has been used to discriminate toxin producing strains in contaminated maize [35]. Whole genome sequencing (WGS) has been employed also for characterization of mycotoxin-producing fungi [36]. Recent advances in such technology and associated reduction in processing costs allow these enhanced molecular tools to be focused on the timely detection of specific groups of organisms from fresh material obtained directly from the field [37]. This approach may now be applied to the detection of toxigenic fungi within the soil community.

The objectives of this study were to develop a polyphasic approach for early detection of aflatoxigenic *A. flavus*, based on soil samples available from seven locations within Pakistan. ITS rRNA metabarcoding was carried out on these samples collected from targeted locations based on production and cultivation. Selected districts in Punjab and Sindh were considered for this focused baseline study. In Punjab, the districts of Okara, Kasur, and Chiniot were selected for sampling; whereas in Sindh, the districts of Mirpurkhas, Matiari, and Tando Allah Yar were chosen. In addition, low coverage WGS was performed on indigenous strains of *A. flavus* isolated from infected fruits as follows: fresh chilies and one-year-old corn cobs to further validate the ITS metabarcoding. Further tests were performed on these indigenous strains of *A. flavus*, including a presumptive culture-based assay for aflatoxin production and parallel generation of MALDI-ToF-MS profiles. This approach may be employed to reduce the potential impact of post-harvest disease and aflatoxins thereby assisting plant health regulators and exporters of agricultural commodities.

## 2. Materials and Methods

### 2.1. Soil Samples Collection and DNA Template Preparation

Soil samples for microbial community characterization were collected from seven locations in different regions of Pakistan (Table 1). 

Soil samples were collected between November 2017 and mid-January 2018, at a depth of 0–20 cm and with a soil corer with a diameter of 1 cm. By sampling at this time, we aimed to test our baseline approach and its applicability for screening agricultural soil for the detection of problematic Aspergilli. This could then be used to determine the ability of these organisms to overwinter in sufficient numbers to be perceived by the methods used in this polyphasic study. Three replicates of each sample were collected within a distance of 10 cm from each other and sent to CAB International (CABI), Egham for testing. Samples were obtained from maize-growing (‘mz’) and chili-growing (‘ch’) soils. Broad details of relevant soil types were obtained from the online ‘Types of soil in Pakistan’ list [38]. DNA was isolated with the DNeasy PowerSoil Kit (Qiagen, Manchester, UK) from 0.5 g of soil, according to the manufacturer’s instructions. After isolation, the DNA quality and quantity were assessed with a Tapestation 4200 (Agilent Technologies Ltd., Stockport, UK) and a Qubit™ 3.0 Fluorometer (Thermo Fisher Scientific, Loughborough, UK), respectively, and normalized to 5 ng/µL for the subsequent PCR reactions.

### 2.2. Visual Assessment of Isolates and Coconut Medium Test

Six field isolates that conformed, morphologically, to *A. flavus* were isolated from corn grains from a cob collected in Burewala. The samples were submitted to CABI’s Diagnostic Advisory Service for investigation and were treated as follows: a one-year-old corn cob, with shrunken kernels and visible black spots, was photographed. The cob was then visually assessed using a model M8 binocular microscope (Wild Heerbrugg, Heerbrugg, Switzerland). Some fungal growth was visible between the kernels and black spots were present on the surface. Individual kernels were removed, of which, four were placed in a damp chamber and incubated on the bench whilst eight were rehydrated for 30 min, surface-sterilized with NaOCl (5%; *v/v*) for 4 min, washed in sterile distilled water, plated out onto two tap water agar plates (TWA; [39]), and incubated at room temperature for up to seven days. Some of the sparse surface mycelium was picked from the cob with a sterile needle and plated out onto TWA. The remainder of the cob was placed in a damp chamber and incubated as described above. Fungal colonies were picked from the isolation/incubation plates as they grew and plated out onto distilled water potato carrot agar (DWPCA; [39] modified to use distilled water rather than tap water) and Czapek yeast autolysate agar (CYA; [39]). Fourteen cultures were isolated and examined morphologically, as follows: a total of 12 originated from the surface sterilized kernels and two from the surface mycelium. Subsequent morphological examination showed six suspected *A. flavus* isolates and two suspected *A. niger* isolates.

Three fresh maize cobs with no obvious disease symptoms were investigated. Samples were photographed, then visually assessed using the binocular microscope as described above. The cob exhibited no visible signs of fungal growth and appeared to be healthy. Individual kernels were removed and treated as described above. The remainder of the cob was placed in a damp chamber and incubated on the bench. Fungal colonies were picked from the isolation/incubation plates as they grew and plated out onto fresh DWPCA. Three cultures were isolated from the surface sterilized kernels and were examined morphologically: it was clear that no *Aspergillus* sp. had been isolated.

The strains were subcultured into Pitt’s medium, and used to prepare a spore suspension. For the fluorescence-based determination of aflatoxin production, 5 µL of spore suspension of each isolate of *A. flavus*, plus three positive controls and three negative controls, were each inoculated onto three plates of coconut milk agar (CMA). CMA was prepared using 200 mL of commercial coconut milk, 600 mL of distilled water (pH 6.9), and 16 g of agar, and autoclaved for 10 min at 121 °C [40]. Small sections of fruit were dissected and placed in damp chambers onto sterile petri dishes to facilitate fungal growth. Other sections of fruit were washed in sterile water and plated onto TWA and additional sections were surface-sterilized and placed on TWA. Fungal colonies, generated from these plates, were removed and placed on DWPCA plates. Pure cultures were identified by morphological examination after 5 to 7 days of incubation in an inverted position in the dark at 28 °C. The bottoms of the plates were exposed to UV light (365 nm); colonies that showed fluorescence were positive (+), while those without fluorescence were negative (−).

### 2.3. MALDI-ToF-MS

The subcultures of the ex-corn *A. flavus* strains, obtained as described below (Section 2.5), were investigated by matrix-assisted laser-desorption and ionization time-of-flight mass spectrometry (MALDI-ToF-MS [41]). The mycelium was precipitated for 1 min at 10,000 x g in a Minispin Plus microcentrifuge (Eppendorf, Stevenage, UK, and 50 μL of MALDI Reagent #1 (11 mg/mL C2020 α-cyano-4-hydroxycinnamic acid (HCCA; ≥98% purity), 65% (*v/v*) acetonitrile, 2.5% (*v/v*) trifluoroacetic acid (TFA), and 32.5% (*v/v*) water) (all components of MALDI Reagent #1: Sigma Aldrich Co, Gillingham, UK) was added to the pellet. The biomass was disrupted in the above reagent using the blunt end of a plastic plating loop. The mixture was vortexed briefly and centrifuged for 1 min at 10,000× *g*. An aliquot of 1 μL of the resulting supernatant was placed onto the MBT Biotarget 96 sample plate (Bruker, Coventry, UK), air dried, and loaded into a linear mode mass spectrometerer (Bruker).

### 2.4. ITS rRNA Metabarcoding

The primer pair ITS1Fl2/ITS2 contained the adapter overhang nucleotide sequences to the 5′-end (Illumina, Cambridge, UK), and was selected for amplification. Libraries were prepared according to the metagenomics sequencing library preparation protocol (Illumina) as described in the manufacturer’s instructions. Primer sequences were as follows: ITS1 Fl2 5′-GAACCWGCGGARGGATCA-3′ [42]; ITS2 5′-GCTGCGTTCTTCATCGATGC-3′ [43]. ITS1FI2 overlaps in six positions with ITS1F [43], but is located closer to the end of the 18S and was specifically designed to improve the understanding of the community composition and distribution of complex communities by achieving deeper sequencing. PCR was undertaken in a Mastercycler Pro S vapo.protect thermal cycler (Eppendorf) with a reaction mix containing 5 µL of each primer at the concentration of 1 µM, 2.5 µL of template DNA at the concentration of 5 ng/µL, and 12.5 μL of KAPA HiFi HotStart ReadyMix (Roche Life Sciences, Welwyn, UK) to a final volume of 25 μL with PCR grade water. PCR reactions were preincubated for 3 min at 95 °C followed by 25 cycles of 30 s at 95 °C, 30 s at 55 °C, and 30 s at 72 °C. Samples were finally incubated for 5 min at 72 °C, followed by chilling to 10 °C. Aliquots of 1 μL of amplification products were assessed for quality with an Agilent Tapestation 4200 (Agilent Technologies Ltd.). A clean-up was undertaken on PCR products with AMPure XP beads (Beckman-Coulter, High Wycombe, UK), following the manufacturer’s instructions. Purified products were resuspended in 25 μL of 10 mM Tris-HCl pH 8.5, diluted from TRIS-HCl pH 8.5; 0.1 M – Ethanol 20% (*v/v*) solution (Sigma Aldrich Co). Index PCR was carried out on purified PCR products for the attachment of sequencing dual indices with the Nextera XT Index Kit (Illumina). The PCR reaction was undertaken in an Mastercycler Pro S vapo.protect with a reaction mix containing 5 µL of template purified PCR product, 5 µL of Nextera XT Index-1, 5 µL of Nextera XT Index-2, 25 μL of KAPA HiFi HotStart ReadyMix, and to a final volume of 50 μL with PCR grade water. The reactions were preincubated for 3 min at 95 °C followed by 8 cycles of 30 s at 95 °C, 30 s at 55 °C, and 30 s at 72 °C. Samples were finally incubated for 5 min at 72 °C, followed by chilling to 10 °C. A second clean-up was carried out on the above products with AMPure XP beads, following the manufacturer’s instructions. Purified products were resuspended in 50 µL of 10 mM Tris-HCl pH 8.5 (prepared as before). Aliquots of 1 μL of index PCR products were assessed for quality with a Tapestation 4200 (Agilent) and quantified on a Qubit™ 3.0 Fluorometer (Thermo Fisher Scientific). DNA concentration, in nM, was calculated based on the size of DNA amplicons as determined by Tapestation 4200 (Agilent). Concentrated libraries were diluted to 4 nM with 10 mM Tris-HCl pH 8.5 (prepared as above), and aliquots of 5 μL of each library were pooled. Samples were denatured by adding 5 μL of pooled DNA libraries to 5 μL of 0.2 N NaOH and incubated for 5 min at room temperature. Denatured single-stranded DNA was then diluted to a final concentration of 15 pM with the Hybridization buffer (Illumina). In addition, this denaturation step was carried out on 15 pM PhiX control (Illumina). Finally, 30 μL of denatured PhiX control library (Illumina) and 570 μL of denatured amplicon library were combined in a microcentrifuge tube. This ratio determined a 5% PhiX control in the mix. The combined library and PhiX control tube were incubated at 96 °C for 2 min, mixed, and placed in an ice/water bath for 5 min. The mix was then loaded into a V3-600 cycles sequencing kit (Illumina) and transferred to the MiSeq (Illumina) for sequencing.

### 2.5. Whole Genome Sequencing

A loopful of spore suspension was transferred on to the agar surface of a distilled water malt agar (MADW) [44] petri dish, in three replicates, to form a triangular arrangement of the inocula. The plates were incubated at 25 °C until a biomass was obtained to fill half of the Petri dish. After seven days of incubation, the isolates were subcultured into sterilized liquid Yeast Malt Broth (YMB, 0.5% (*w/v*) peptone, 0.3% (*w/v*) yeast extract, 0.3% (*w/v*) malt extract, 1% (*w/v*) glucose in deionized water). A portion of MADW medium of each subculture was taken from the edge of actively growing colonies and transferred into 1 mL of sterile water. The mycelium was disrupted with a micropestle for the inoculation into universal tubes. Universal tubes containing 10 mL of liquid YMB were used to generate the preinoculum, after incubation in the dark in an orbital shaking incubator (Gallenkamp, Weiss Technik, Loughborough, UK) at 25 ± 2 °C at 100 rpm for 1 day. At the end of the incubation period, the contents of each universal tube (preinoculum) were transferred into 250 mL Erlenmeyer flasks containing 100 mL of YMB and incubated in the dark at 25 ± 2 °C at 150 rpm for 2 days. The biomass from each flask was recovered by vacuum filtration onto Whatman No. 3 filter paper (GE Healthcare Life Sciences, Amersham, UK) and stored in a sterile Petri plate at −20 °C. The mycelium of each sample was pulverized using a sterile mortar and pestle, with 0.035–0.040g of ground mycelium transferred into sterile 2 mL microcentrifuge tubes and stored at −20 °C. Genomic DNA (gDNA) was fragmented after quantification and quality assessment for library preparation. Two hundred ng of gDNA were diluted in 130 µL of 10 mM Tris-HCl pH 8.5 (prepared as above), and sheared with a M220 Focused-ultrasonicator (Covaris Ltd., Brighton, UK) with the following settings for a 550 bp insert size: Duty factor (%) 20, peak power 50.0, cycles/burst 200, and duration of 25 s at 20 °C. Libraries were prepared with a Truseq Nano DNA Library prep kit (Illumina), according to the manufacturers’ instructions. The paired end reads were generated with the MiSeq (Illumina) at CABI (Egham, UK), with an average insert size of 350 bp and run on a V3-600 cycles sequencing kit (Illumina).

### 2.6. Data Analysis

Metabarcoding ITS rRNA data were analyzed by the CABI metabarcoding pipeline. This included isolation of ITS1 region by ITSx [45], and quality control of DNA reads. The threshold to accept OTUs as present in a sample was set to 0.005, thereby requiring 0.5% of mapped reads per sample to be assigned to an OTU for that OTU to be considered truly present in the given sample. For WGS, high quality reads were filtered from raw reads with the MiSeq Reporter analysis software v3.0 (Illumina). Assembly was performed with SPAdes v3.11.1 [46], and the annotation of the complete mt genome was performed with GeSeq [47]. The circular mt genome maps were generated with the online tool OGDraw v1.2 [48] with default settings using *A. flavus* NC_026920 as reference genomes. Partial barcode sequences were aligned using Bioedit v7.2.6.1 (Isis Pharmaceuticals, Carlsbad, CA, USA). Datasets were submitted to the NCBI SRA Database with the accession number: PRJNA550330.

## 3. Results

### 3.1. ITS rRNA Metabarcoding of Maize and Chili Soils

A total number of 2,525,524 ITS1 rRNA reads were obtained from the soil samples. Operational taxonomic units (OTU) were quality filtered and those that were at least >1% of the total abundance were kept for further investigations (Table 2).

**Figure 1:**
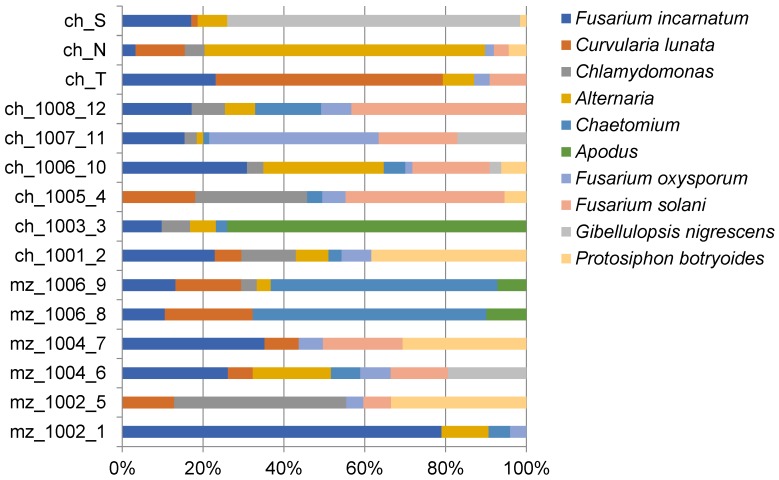
ITS rRNA metabarcoding showing the 10 most abundant operational taxonomic units (OTU) from the study plotted against each soil sample, showing trends in inter-sample relative abundance.

Of the total of 179 OTU found in at least one sample with >1% of DNA reads, the ten most abundant OTU were selected and plotted against each soil sample (Figure 1). 

Major changes in relative abundance were observed for *Fusarium incarnatum*, for which high relative quantities were of 16.4%, 12%, and 11.3% in mz_1002_1, ch_1006_10, and “S”, respectively. *Chaetomium* sp. was detected with relative abundance >10% in two maize soils (13.8% and 11.7% in 1006_8 and 1006_9, respectively) and *Alternaria* sp. was recovered at 11.5% and the 27.8% of fungal diversity in chili soils 1006_10 and “N” respectively. *Fusarium oxysporum* was found at 20.4% of chili 1007_11, *Curvularia lunata* represented 10.9% in chili “S”, and the soil alga *Protosiphon botryoides* was found at 10.1% Maize 1002_5. Uncultured *Apodus* sp. and *Gibellulopsis nigrescens* were detected in large relative abundance in chili soil (28.8% in 1003_3 and 48.8% in “S”).

### 3.2. Trends of Aspergillus flavus (OTU62) in the Soil

Putative aflatoxigenic *Aspergillus flavus* was detected in four of the 15 soil samples investigated and represented by OTU62 as follows: Three chili soil samples (ch_1005_4, ch_1007_11, ch_T) and one maize soil (mz_1004_6). 

**Figure 2:**
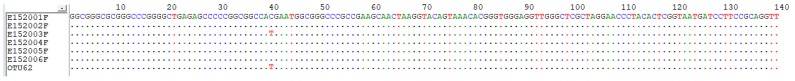
Alignment (using Bioedit v7.2.6.1) of partial ITS1 region of the ribosomal cluster of the six strains of *A. flavus*, against OTU62.

When found, OTU62 was always below 2% of the total DNA reads, with a peak of 1.51% of relative abundance for sample ch_1005_4. The remaining 11 soils did not show evidence of the presence of OTU62. Additional investigations were undertaken on OTU62. Alignment of OTU62 against the ribosomal cluster generated by whole genome sequencing (WGS) (see Section 3.5) confirmed that the rRNA detected in the four soils was identical to that of the aflatoxigenic strain E152003F (Figure 2).

### 3.3. Characterisation of A. flavus Strains by Coconut Medium Test

**Figure 3:**
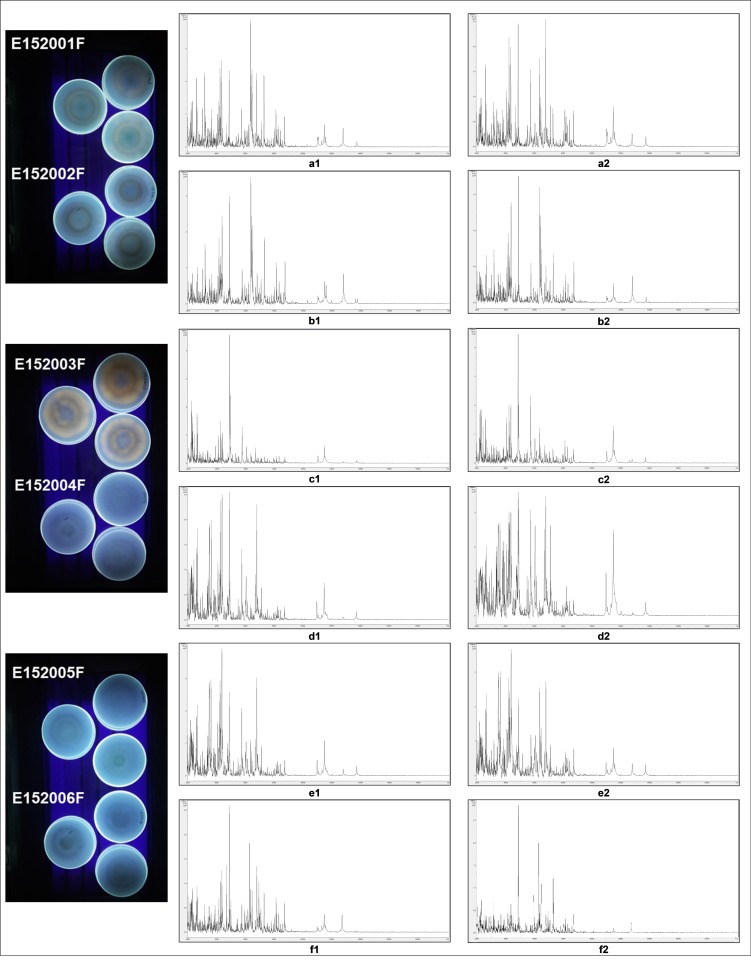
Visualization of coconut medium test under UV light, and the MALDI-ToF-MS duplicate spectra of putative *Aspergillus flavus* strains E152 (001F-006F): E152001F (a1, a2), E152002F (b1, b2), E152003F (c1, c2), E152004F (d1, d2), E152005F (e1, e2), E152006F (f1, f2). Duplicate spectra are shown baseline-subtracted, smoothed, y-axis-autoscaled, and covering the mass range 2 kDa to 20 kDa (with x-axis scale increments of 2 kDa). (Duplicate spectral profiles (from separate extractions ‘1′ and ‘2′) are shown for each isolate, a–f. Further information may be found in the Supplementary Materials).

The six strains of putative *A. flavus* were isolated and identified by ITS rRNA molecular sequencing, and further investigated by a coconut medium test. Aflatoxigenic strains were expected to have a fluorescent reaction under UV light (254 nm). Strain E152003F was the only isolate to produce a fluorescent reaction with this CMA test (Figure 3). Fungal growth was observed on chilies, which appeared to be otherwise healthy. Surface fungi on chili were picked off with a sterile needle and placed on PCA plates. Based on morphological identification, six strains of *A. flavus* (E152001F-006F) were selected from the corn cobs, while no *A. flavus* strains were isolated from chili. 

### 3.4. MALDI-ToF-MS of Aspergillus flavus Strains

Further tests were undertaken on the six strains of *A. flavus*. Pure cultures were subjected to MALDI-ToF-MS analysis (Figure 3). Strain E152003F gave a unique spectral profile, whilst the other strains gave a characteristic profile for the group. Within this group, strains E152004F and E152005F were very similar to each other, as were E152002F and E152006F. It was noted that E152004F was less similar to E152002F and to E152006F than they were to each other. This suggests that the group may contain two subgroups (E152004F and E152005F) and (E152002F and E152006F); while E152001F was distinct from both subgroups. Pairwise comparison of spectral profiles from the duplicate samples are given in the Supplementary Materials.

### 3.5. Whole Mitochondrial Genomes and Nuclear Ribosomal Cluster of A. flavus

**Figure 4:**
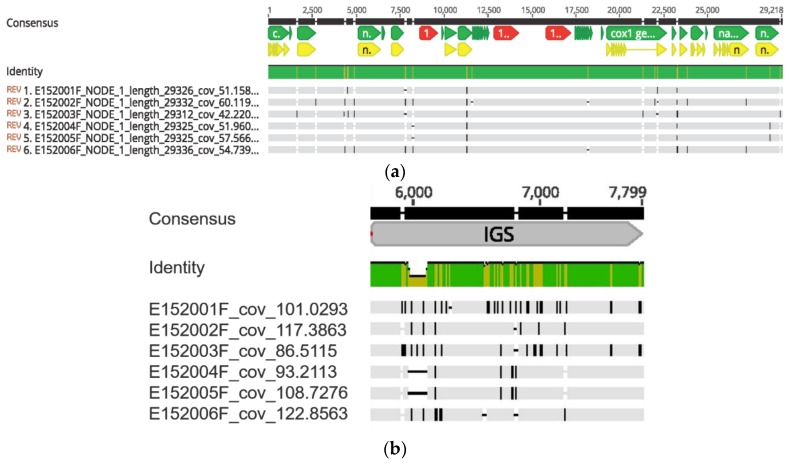
Aligned mitochondrial genome of the six *A. flavus* strains isolated from infected corn cob (**a**). Aligned IGS region of the ribosomal cluster of the six *A. flavus* strains isolated from infected corn cob (**b**).

The six strains of *A. flavus* (E152, 001F-006F) were further investigated by whole genome sequencing (WGS). DNA reads were reassembled and generated high coverage contigs for the whole ribosomal cluster (rRNA) and the whole mitochondrial (mt) genomes (Figure 4a). The highest variability within the ribosomal clusters was seen in the IGS region (Figure 4b).

**Figure 5:**
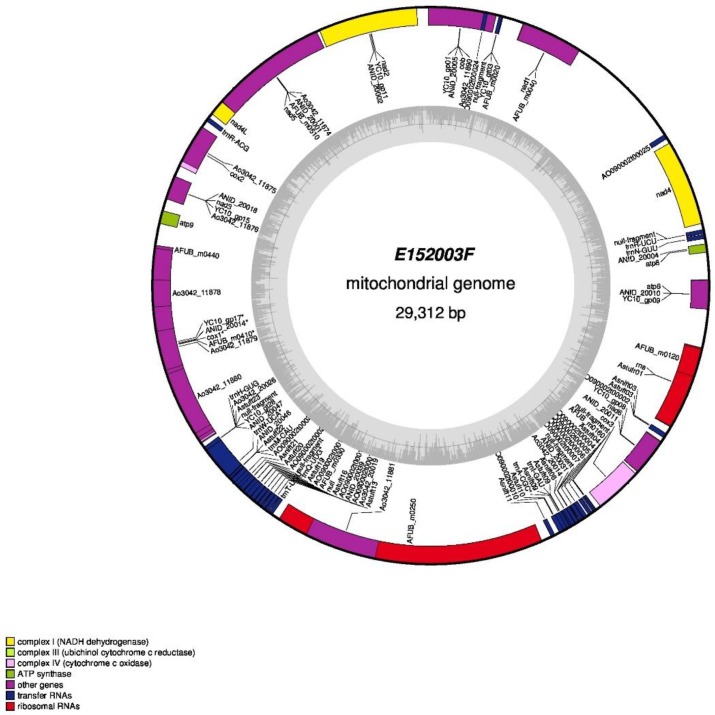
Gene map of the mitochondrial DNA (mtDNA) of *A. flavus* strain E152003F. The colored bars indicate different functional groups. The dark grey area in the inner circle corresponds to GC content while the light grey corresponds to the adenine-thymine (AT) content of the genome.

Strains E152004F and E152005F showed identical rRNA cluster and mt genome sequences, while E152001F and E152002F were similar, with some differences in the IGS region of the rRNA (Figure 4b). On average, the coverage of the ribosomal clusters of the six strains was of 104.95X and the coverage of the mt genomes of the six strains was 52.9X. The strain E152003F gave unique IGS region of the ribosomal cluster (Figure 4b) and the mt genome (Figure 5). The mt genome of E152003F was further analyzed by annotation and showed a typical structure consistent with that of Ascomycota. The final mt genome could be represented as a circular molecule, and was of 29,312 bp. The genome contained 12 protein-coding genes (PCG), 27 transfer RNA, and three ribosomal RNA genes.

## 4. Discussion

Successful isolation and identification of aflatoxigenic *A. flavus* was demonstrated by a combination of culture based and molecular methods. Eight isolates were obtained from a one-year-old corn cob that displayed symptoms consistent with *Aspergillus* colonization. Seven of these eight *Aspergillus* strains were identified as follows: two were *A. niger*, five were *A. oryzae* or *A. flavus*. The remaining strain, whilst conforming to *A. flavus* morphologically, did not produce a readable sequence, thereby suggesting that it may have been contaminated by a secondary organism. No *Aspergillus* spp. were isolated from the tested chilies. The six suspected *A. flavus* strains (E152001F-006F) were further investigated and evidence of the aflatoxigenic biotype was seen in sample E152003F. In order to support these findings, we undertook a culture-based assay for aflatoxin production using the coconut milk medium assay of Fente and co-workers [40]. Aflatoxigenic strains were expected to have a fluorescent reaction under UV light. This method has been employed successfully in a previous polyphasic analysis [10]. The strain E152003F was the only one to produce a fluorescent reaction. In addition, MALDI-ToF-MS was carried out on the six *A. flavus* strains. Strain E152003F gave a unique spectral profile, whilst the remaining five isolates shared a common profile.

The six *A. flavus* strains (E152001F-006F) were subjected to Whole Genome Sequencing (WGS) with the MiSeq (Illumina). This approach was undertaken in this baseline study to achieve the maximum information within the timescale and funding available. This required an innovative approach using WGS with low coverage in order to obtain the whole ribosomal cluster (rRNA) and the whole mt genome. The majority of the genetic variability of the rRNA for these *A. flavus* strains was confined to the IGS region. Strain E152003F showed a unique sequence thereby supporting the findings above. Likewise, alignment of mt genomes across the strains confirmed this result.

Four of the 15 soil sample sets investigated contained the putative aflatoxigenic *A. flavus* genotype (OTU62), as shown by a comparison of results from ITS metabarcoding of the soil samples with those from ITS barcoding of pure cultures tested on Coconut Milk Agar (CMA). As soil samples are extremely complex environments, with taxa present at many trophic and functional levels and covering microorganisms, plant tissue, and meso- and macrofauna, molecular tools may be used to discriminate at the functional and/or the taxonomic level. Rather than screen for aflatoxin genes by targeted diagnostic methods, e.g., qPCR [10], or fingerprinting methods, e.g., RAPD [28], AFLP [29], and microsatellite markers [30], we opted to gain sequence information regarding the composition of the soil fungal microbiome through a metabarcoding approach. Combining the metabarcoding with isolation of strains from the soil validated the identity of the relevant OTU in the soil, therefore giving added confidence to the inferences made. 

Whilst identifications in the genus *Aspergillus* cannot be resolved to species-level with the recognized fungal barcode of ITS (e.g., Reference [25]), we used the results of our polyphasic analysis to determine a putative aflatoxigenic ITS OTU that could be employed in our metabarcoding study. Recovery of DNA from fungi may be affected by the relative life stage, structure, or size present in the environment, for example some fungal spores may not conducive to all DNA isolation methods [49,50]. This rRNA metabarcoding revealed a low relative abundance of *A. flavus* OTU62 in the soil, indicating that there exists the potential for mycotoxigenic contamination of the crop at a later stage. Such findings support the hypothesis that soils where maize and chili are grown may act as reservoirs for aflatoxigenic *Aspergillus* spp. This also suggests the potential for further development of aflatoxigenic *A. flavus* at a later stage, e.g., post-harvest. Low incidences were recorded for all *Aspergilli* also, as they were not present in the list of the top 10 most abundant OTU, showing that such methods that only target the most abundant soil inhabitants may miss low level sources of future infection [51]. One outcome from microbiome investigation is the recognition that future biological control programs should incorporate pre- and post-intervention screening of the microbiome with appropriate control plots and place.

Whilst we appreciate that this was only a relatively limited baseline study, it is the first time that the present approach has been used. Previous polyphasic investigation has shown that even a well targeted aflatoxin gene primer set cannot guarantee results correlated with aflatoxigenicity [10]. A definitive species-level identification of strains within the genus *Aspergillus* requires additional sequencing analysis beyond rRNA, such as calmodulin [52]. Our approach, in a manner similar to Rodrigues et al. [10], was to apply a polyphasic methodology which combined next generation sequencing with a classical agar plate-based test to validate rRNA metabarcoding, low coverage WGS, and the coconut milk agar study, then ITS rRNA barcoding. We did not use single copy nuclear genes, such as those of the aflatoxins gene cluster [10], because of the resolution of our modified polyphasic approach. This, in addition to the coconut medium test (CMA test), generated complete mt genomes and full ribosomal clusters of indigenous strains, which is where it differs from previous research [10]. The ribosomal clusters showed the highest intra-specific diversity in the IGS region; this region would not be appropriate for metabarcoding due to its high levels of infra-specific variability. Such heterogeneity would make it very difficult to create ‘universal’ primers for broad application.

The alignment of the rRNA clusters show clear differentiation between aflatoxigenic and non-toxigenic strains (as shown by the CMA assay), thereby aiding successful ITS rRNA metabarcoding. The four methods employed in this study (a) ITS rRNA metabarcoding, (b) low coverage WGS, (c) the CMA test, and (d) MALDI-ToF-MS, when combined, became considerably more powerful in their resolving capability, whilst each would not be conclusive and/or informative if used on its own.

This study enabled a cost-effective approach for screening agricultural soils for potentially aflatoxigenic Aspergilli, and could be extended to other groups of soil-borne mycotoxigenic fungi. Adoption of an annual and/or seasonal monitoring program would facilitate tracking and trending of microbial groupings, and particularly of toxigenic genotypes, to be undertaken. Implementation of this approach, which may allow early detection of microbial contamination whilst reducing processing costs, could enhance informed decision making by growers and plant health authorities in Pakistan.

## Figures and Tables

**Table 1:** Soil samples collected for the baseline study (with, respectively, reference number: ‘mz’ prefix = maize growing, ‘ch’ prefix = chili growing soil, soil type [38], location (nearest town), date of collection, and geospatial coordinates).

Reference	Soil Type	Location	Date of Collection	Geospatial Coordinates
mz_1002_1	Silt-loam	Okara	29/12/2017	30.801380° N, 73.448334° E
mz_1002_5	Silt-loam	Okara	29/12/2017	30.801380° N, 73.448334° E
mz_1004_6	Silt-loam	Kasur	29/12/2017	31.11866° N, 74.4502487° E
mz_1004_7	Silt-loam	Kasur	29/12/2017	31.11866° N, 74.4502487° E
mz_1006_8	Sandy-loam	Chiniot	30/12/2017	31.72° N, 72.97889° E
mz_1006_9	Sandy-loam	Chiniot	30/12/2017	31.72° N, 72.97889° E
ch_1001_2	Silt-loam	Okara	29/12/2017	30.801380° N, 73.448334° E
ch_1003_3	Silt-loam	Kasur	29/12/2017	31.11866° N, 74.4502487° E
ch_1005_4	Sandy-loam	Chiniot	30/12/2017	31.72° N, 72.97889° E
ch_1006_10	Silty/clay-loam	Mirpurkhas	14/01/2018	25.5251° N, 69.0159° E
ch_1007_11	Loamy-soil	Tando Allah Yar	14/01/2018	25.46263° N, 68.71923° E
ch_1008_12	Loamy-soil	Matiari	15/01/2018	25.59609° N, 68.44666° E
ch_T	Sandy to clay loam	Sheikhupura	01/11/2017	31.7167° N, 73.9850° E
ch_N	Sandy to clay loam	Sheikhupura	01/11/2017	31.7167° N, 73.9850° E
ch_S	Sandy to clay loam	Sheikhupura	01/11/2017	31.7167° N, 73.9850° E

**Table 2:** ITS rRNA raw reads and diversity indices per sample.

Sample ID	Number of Reads	Number of OTU
mz_1002_1	153,684	39
mz_1002_5	173,931	42
mz_1004_6	161,120	45
mz_1004_7	107,104	33
mz_1006_8	234,939	34
mz_1006_9	267,858	41
ch_1001_2	136,776	44
ch_1003_3	158,472	33
ch_1005_4	108,841	53
ch_1006_10	141,503	37
ch_1007_11	205,729	29
ch_1008_12	35,556	47
ch_T	231,006	37
ch_N	208,840	29
ch_S	200,165	17

## Supplementary Materials

The following are available online at https://www.mdpi.com/2076-2607/7/9/300/s1.
