# Supplementary material for: A Polyphasic Approach Aids Early Detection of Potentially Toxigenic Aspergilli in Soil
Source: Microorganisms. 2019 Aug 29;7(9):300. doi: 10.3390/microorganisms7090300 (PMC6781248; doi:10.3390/microorganisms7090300)
Supplement: Supplementary file 1 [file microorganisms-07-00300-s001.pdf]

All sequence data are available on request. Original spectral data held on the Microflex PC (Bruker) are available on request.

Supplementary materials

Replicate spectra (designated A and B) were generated for each sample according to the methods described in section 2.6 of the manuscript. The B sample profiles were used to make a spectral database against which the A sample spectra were screened. Best and second best matches for each sample are presented below:

AnalyteName	Organism(best match)	ScoreValue	Organism(second best match)	ScoreValue
E152002_A	E152002_B	2.71	E152006_B	2.338
E152001_A	E152001_B	2.578	E152002_B	2.466
E152003_A	E152003_B	2.68	E152001_B	2.075
E152005_A	E152005_B	2.603	E152004_B	2.578
E152004_A	E152004_B	2.666	E152005_B	2.515
E152006_A	E152006_B	2.687	E152002_B	2.372

Meaning of Score Values

Range	Description	Color
**2.300 ... 3.000**	**highly probable species identification**	green
**2.000 ... 2.299**	**secure genus identification, probable species identification**	green
**1.700 ... 1.999**	**probable genus identification**	yellow
**0.000 ... 1.699**	**not reliable identification**	red

Result: the best matches are all against the same strain. This means that the strains are all slightly different from each other in terms of their spectra. The scores are all very high (above 2.3) so these are high-confidence matches and indicate that the spectra are all of high quality.

The second best matches are all above 2.3 except for one. This means that most of the samples are very similar except for sample E152003, which only just scrapes in as a green match above 2.0. This suggests that sample E152003 is less closely related to the others.

Individual analyses

E152002_A

Rank(Quality)	Matched Pattern	ScoreValue
1	E152002_B	2.71

2	E152006_B	2.338
3	E152001_B	2.266
4	E152005_B	2.162
5	E152003_B	1.955
6	E152004_B	1.54

E152001_A

Rank(Quality)	Matched Pattern	ScoreValue
1	E152001_B	2.578
2	E152002_B	2.466
3	E152005_B	2.19
4	E152003_B	2.151
5	E152006_B	2.11
6	E152004_B	1.711

E152003_A

Rank(Quality)	Matched Pattern	ScoreValue
1	E152003_B	2.68
2	E152001_B	2.075
3	E152004_B	2.025
4	E152002_B	1.913
5	E152005_B	1.875
6	E152006_B	1.836

E152005_A

Rank(Quality)	Matched Pattern	ScoreValue
1	E152005_B	2.603
2	E152004_B	2.578
3	E152001_B	2.192
4	E152003_B	2.15
5	E152002_B	1.988

6	E152006_B	1.9

E152004_A

Rank(Quality)	Matched Pattern	ScoreValue
1	E152004_B	2.666
2	E152005_B	2.515
3	E152001_B	2.197
4	E152003_B	2.159
5	E152002_B	1.808
6	E152006_B	1.655

E152006_A

Rank (Quality)	Matched Pattern	Score Value
1	E152006_B	2.687
2	E152002_B	2.372
3	E152001_B	2.26
4	E152003_B	2.09
5	E152005_B	2.053
6	E152004_B	1.582

From the above, E152003 is the spectrum that stands out as not having another strain that is very closely related to it. E152004 and E152005 are very similar to each other and E152002 and E152006 are very similar to each other but E152004 is very dissimilar to E152002 and to E152006 so this argues for two somewhat separate groupings (E152004 and E152005) and (E152002 and E152006), with E152001 most closely related to the (E152002 and E152006) grouping, with E152003 as a relative outlier.
